# Effects of aerobic exercise on approach–avoidance responses and prefrontal activity in individuals with tobacco dependence

**DOI:** 10.3389/fspor.2026.1751338

**Published:** 2026-03-20

**Authors:** Hongen Liu, Yingying Zhang, Bin Zhang, Si Zhang, Yanbai Han, Zhao Xu

**Affiliations:** 1 Guangxi Normal University, Guilin, China; 2 Shandong Sports University, Jinan, China

**Keywords:** aerobic exercise, approach bias, fNIRS, prefrontal cortex, smoking-related cues, tobacco dependence

## Abstract

**Objective:**

This study examined whether smokers exhibit an approach bias toward smoking-related cues and investigated the effects of acute aerobic exercise on modifying this behavioral tendency and its associated prefrontal cortical activity.

**Methods:**

In Experiment 1, a stimulus–response compatibility task was used to compare approach–avoidance behaviors between smokers (*n* = 40) and non-smokers (*n* = 40) under smoking-related and neutral cue conditions. Experiment 2 adopted a randomized controlled design in which 60 smokers were assigned to moderate-intensity exercise, high-intensity exercise, or a resting control group. Participants completed the stimulus–response compatibility task before and after intervention, while prefrontal oxygenated hemoglobin was recorded using fNIRS.

**Results:**

Smokers demonstrated a significant approach bias toward smoking-related cues, and the magnitude of this bias was positively associated with nicotine dependence levels. Acute aerobic exercise significantly altered the cognitive processing underlying this bias, evidenced by reduced behavioral approach bias scores and decreased prefrontal activation in response to smoking cues.

**Conclusion:**

Acute aerobic exercise temporarily strengthens smokers' cognitive control over smoking-related cues, attenuates approach bias, and improves prefrontal cortical function. These findings provide empirical support for incorporating aerobic exercise into smoking cessation interventions.

## 1 Introduction

Tobacco dependence is one of the most prevalent forms of substance addiction, posing substantial risks to both individual health and public well-being. Once absorbed into the body, nicotine abnormally activates neural circuits involved in the processing of addiction-related cues (1), markedly increasing the motivational value of tobacco stimuli. As a result, smoking-related cues more readily capture attention and elicit craving-related responses (2). Behaviorally, this heightened cue reactivity is often expressed as an approach bias toward tobacco cues, whereby smokers display automatic approach tendencies or consumption-related responses when unconsciously exposed to such cues (3, 4). As a stable cognitive bias, approach bias not only underpins the maintenance of smoking behavior but also represents a key target for interventions aimed at reducing tobacco dependence.

Along with the growing recognition of approach bias as a key cognitive mechanism in tobacco dependence, increasing attention has been directed toward its underlying neural basis. The prefrontal cortex (PFC)—including regions such as the dorsolateral, ventromedial, and orbitofrontal areas—plays a central role in executive control, inhibitory processing, reward evaluation, and emotion regulation (5, 6). Evidence from functional magnetic resonance imaging (fMRI) and functional near-Infrared spectroscopy (fNIRS) studies shows that exposure to smoking-related cues often reduces activation in cognitive control regions such as the dorsolateral PFC, while simultaneously increasing activation in reward-related areas including the medial PFC (7–9). This imbalance impairs inhibitory control and strengthens approach tendencies toward tobacco cues. Moreover, individuals with higher levels of nicotine dependence frequently display atypical PFC activation patterns during decision-making and cue-reactivity tasks, suggesting that impaired prefrontal function may serve as a core neural marker of addiction-related approach behavior (10–13). Therefore, assessing PFC activity is essential for understanding the mechanisms underlying approach bias and for elucidating how exercise-based interventions may modulate responses to addiction-related cues.

Exercise, as a non-pharmacological intervention, has been shown to enhance various cognitive functions, including attention and executive control (14, 15). In individuals with tobacco dependence, exercise can effectively attenuate reactivity to smoking-related cues and reduce attentional focus on tobacco stimuli. Furthermore, numerous studies indicate that physical activity significantly decreases nicotine craving, with smokers consistently reporting notable reductions in craving following exercise (16). Aerobic exercise, in particular, not only modulates behavioral responses to smoking cues but may also alter cognitive processing, thereby reducing the motivational value of tobacco-related stimuli (17, 18). These findings suggest that exercise-induced modulation of approach bias toward smoking cues may represent a viable strategy for reducing smoking behavior.

Acute exercise has been increasingly used as an experimental model to examine transient modulations of cognitive control and PFC function. Compared with chronic exercise training, acute exercise induces rapid and reversible changes in prefrontal neurovascular coupling, which can be sensitively captured by fNIRS through changes in oxygenated hemoglobin (HbO) concentration. Previous studies have shown that a single bout of aerobic exercise elicits short-term increases in prefrontal HbO, reflecting enhanced cerebral blood flow and task-related cortical engagement (19, 20), whereas long-term exercise training is more likely to induce cumulative and stable neural adaptations (21). Moreover, compared with resistance exercise, aerobic exercise has been more consistently associated with greater prefrontal hemodynamic responses, likely due to higher cardiovascular load and more efficient cerebral oxygen delivery (22). In contrast, resistance exercise has been reported to induce more variable prefrontal hemodynamic changes, depending on exercise intensity and task demands (23). In this context, acute aerobic exercise provides a well-controlled framework to characterize state-dependent changes in approach–avoidance behavior and PFC activity, while minimizing confounding effects associated with long-term training.

In summary, approach bias toward tobacco cues may represent a key psychological mechanism underlying smokers' difficulty in quitting, warranting particular attention in cessation interventions. Meanwhile, as exercise-based interventions gain prominence in substance dependence research, an increasing number of studies have examined exercise-induced changes in cognitive functions related to reward processing and emotional states (24, 25). However, studies specifically investigating the modulation of approach bias in tobacco-dependent individuals remain limited, especially those combining behavioral measures with prefrontal cortex activity. Accordingly, the present study recruited smokers to examine the effects of acute aerobic exercise on approach bias, and to further assess whether exercise can simultaneously modulate both behavioral responses to tobacco cues and prefrontal cortical activity, thereby providing new evidence to support exercise-based smoking cessation interventions.

## 2 Methods

### 2.1 Participants

#### 2.1.1 Sample size calculation

Sample size estimation was conducted *a priori* using G*Power 3.1. For Experiment 1, assuming an effect size of 0.25, a significance level of *α* = 0.05, and a statistical power of 0.95, the minimum required sample size was 54 participants; a total of 80 participants were ultimately recruited, including 40 smokers and 40 non-smokers. For Experiment 2, under the same assumptions, the estimated minimum sample size was 45 participants, and 60 participants were enrolled. The actual sample sizes were deliberately higher than the minimum requirements to mitigate the potential impact of participant dropout on statistical power, ensuring sufficient power for subsequent analyses.

#### 2.1.2 Participant inclusion criteria

All participants in the study were male. Female smokers were not included due to their relatively low prevalence in the target population, which would have limited the feasibility of recruitment and statistical power. All participants met the following inclusion criteria: (1) normal or corrected-to-normal vision; (2) right-handedness; (3) no history of mental disorders or other substance abuse; (4) Participants had not engaged in any structured or systematic exercise training (e.g., aerobic or resistance training) for at least 6 months prior to enrollment; (5) smokers were selected using the Fagerström Test for Nicotine Dependence (FTND) (26), with FTND scores ≥4 indicating at least moderate nicotine dependence, and (6) non-smokers, no prior history of smoking, including electronic cigarettes. The basic demographic of the participants is summarized in Table 1, and no significant differences were observed between groups in either experiment (*p* > 0.05).

**Table 1:** Basic characteristics of participants.

Group	Experiment 1			Experiment 2			
	Smoker	Non-smoker	*p*	High intensity exercise (n = 20)	Moderate intensity exercise (*n* = 20)	Control	*p*
	(*n* = 40)	(*n* = 40)				(*n* = 20)	
Age(year)	20.83 ± 1.72	20.73 ± 1.88	0.803	20.55 ± 2.16	20.47 ± 2.32	21.10 ± 1.12	0.923
Height(cm)	180.13 ± 5.68	178.95 ± 6.01	0.366	179.8 ± 6.01	178.71 ± 6.56	180.45 ± 5.48	0.686
Weight(kg)	73.24 ± 15.33	75.97 ± 14.37	0.414	74.55 ± 20.12	73.15 ± 9.40	71.93 ± 8.60	0.107
Smoking duration(years)	3.41 ± 1.86			3.00 ± 1.75	3.71 ± 1.96	3.83 ± 1.93	0.680
Cigarettes/day	8.60 ± 4.40			8.95 ± 4.06	9.59 ± 6.42	8.25 ± 4.79	0.065
FTND score	6.58 ± 1.56			6.85 ± 1.84	6.88 ± 1.69	6.60 ± 1.22	0.862

All participants were male.

The study was conducted in accordance with the Declaration of Helsinki and was approved by the Ethics Committee of Shandong Sport University (No. 2022048). All participants provided written informed consent prior to participation.

#### 2.1.3 Randomization and blinding

Experiment 1 adopted a cross-sectional comparative design. Participants were classified into the smoker and non-smoker groups based on standardized nicotine dependence assessment criteria, supplemented by screening procedures. Given the nature of group classification, randomization was not applicable in this experiment. To minimize potential assessment bias, the order of behavioral tasks and stimulus presentation was randomized across participants. Data preprocessing and statistical analyses of data were conducted by investigators who were blinded to participants' group membership.

Experiment 2 employed a randomized controlled design. Eligible smokers were randomly assigned to the high-intensity exercise group, moderate-intensity exercise group, or control group using a computer-generated randomization sequence. Group allocation was concealed until completion of baseline assessments. Due to the nature of the exercise intervention, participants could not be blinded to group assignment. However, outcome assessors and data analysts were blinded to group allocation. The exercise protocols were administered by trained staff following standardized procedures to ensure consistency across participants. Baseline demographic and smoking-related characteristics were comparable across groups, reducing the risk of allocation bias.

### 2.2 Experimental design

**Figure 1:**
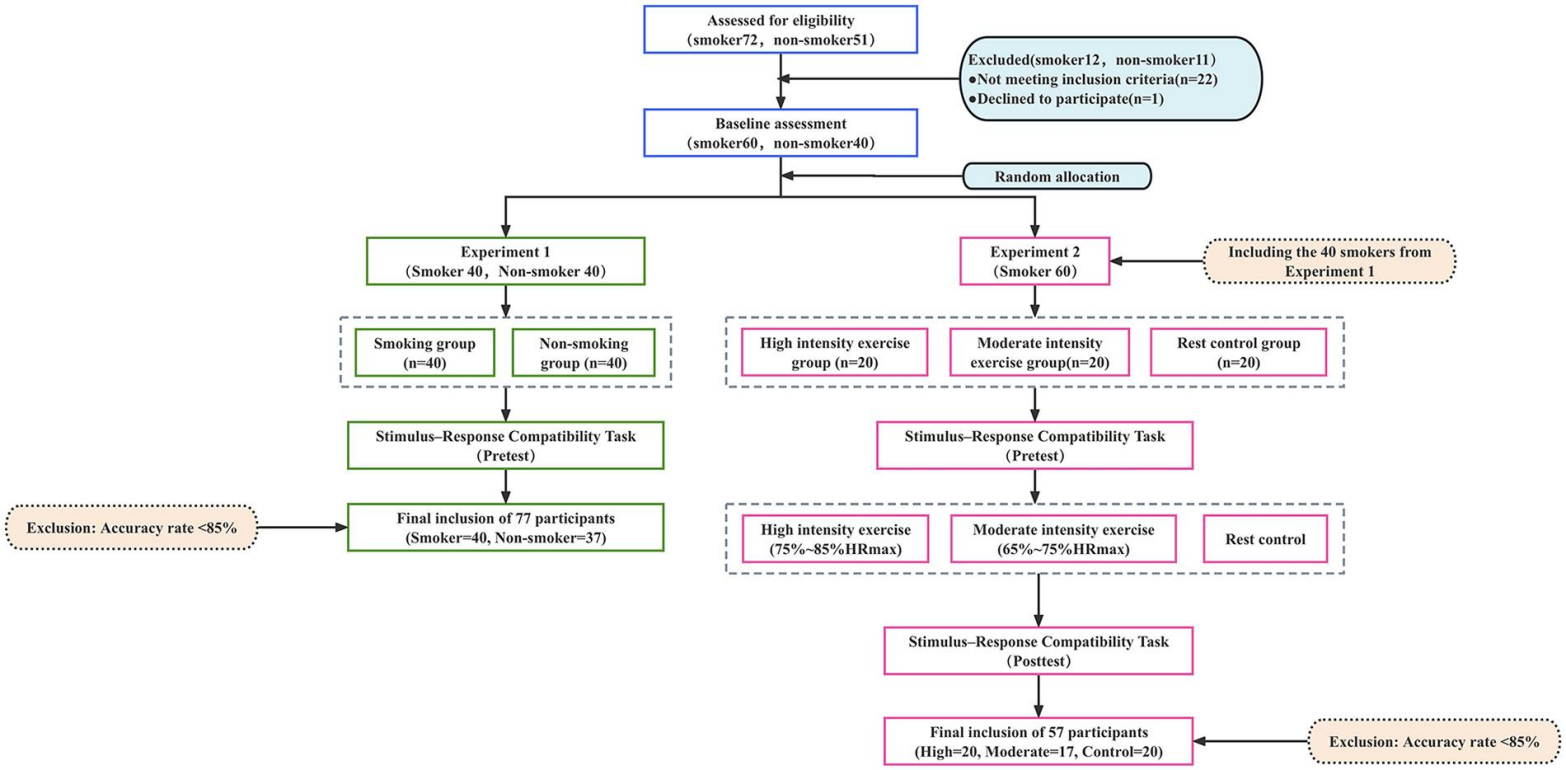
Schematic diagram of study design.

Experiment 2 adopted a mixed factorial design with 3 (Group: moderate-intensity exercise, high-intensity exercise, resting control) × 2 (Stimulus Type: smoking images, neutral images) × 2 (Time: pre-test, post-test). All testing and interventions were completed on the same day. Upon arrival at the laboratory, participants were familiarized with the experimental environment, aerobic exercise equipment, and experimental procedures, and completed a brief practice session of the stimulus–response task to ensure task comprehension (Figure 1).

The pre-test was administered prior to the intervention. Participants then underwent the assigned intervention: both exercise groups completed 35 min of aerobic exercise, whereas the resting control group remained seated quietly in the laboratory for the same duration. To minimize additional cognitive or emotional stimulation, control participants were instructed to remain awake and relaxed without engaging in any cognitive activities such as reading or using electronic devices. To reduce potential environmental influences, exercise and control sessions were conducted in the same laboratory but scheduled at different times. The post-test was conducted immediately after the intervention. During both the pre-test and post-test, participants completed the stimulus–response task while oxygenated hemoglobin concentration in the prefrontal cortex was simultaneously recorded using fNIRS. The entire experimental session lasted approximately 90 min per participant.

Prior to testing, participants were instructed to avoid vigorous physical activity and to refrain from alcohol, strong tea, or caffeine for at least 24 h. Smokers were also asked to abstain from smoking for a minimum of 2 h before the experiment to minimize the acute effects of nicotine while avoiding significant withdrawal symptoms.

### 2.3 Experimental materials

The experiment used 40 smoking images (e.g., lighters, ashtrays, people smoking) and 40 neutral images (e.g., landscapes, people, food, or other scenes without smoking elements), with each image presented twice. All images were obtained from open-access sources available on the Chinese internet. An image evaluation task was conducted using Question Star, with a 1–10 rating scale. Thirty smokers and thirty non-smokers rated each image for clarity and smoking relevance (Table 2). These 60 participants did not take part in the main experiment.

**Table 2:** Evaluation of image clarity and smoking relevance.

Measure	Smoking images		Neutral images	
	Smokers	Non-smokers	Smokers	Non-smokers
Smoking relevance	9.78 ± 0.42	9.81 ± 0.37	1.36 ± 0.29	1.33 ± 0.40
Clarity	9.32 ± 1.03	9.40 ± 0.94	9.15 ± 0.81	9.25 ± 0.98

### 2.4 Procedure and measures

#### 2.4.1 Stimulus-response compatibility task

**Figure 2:**
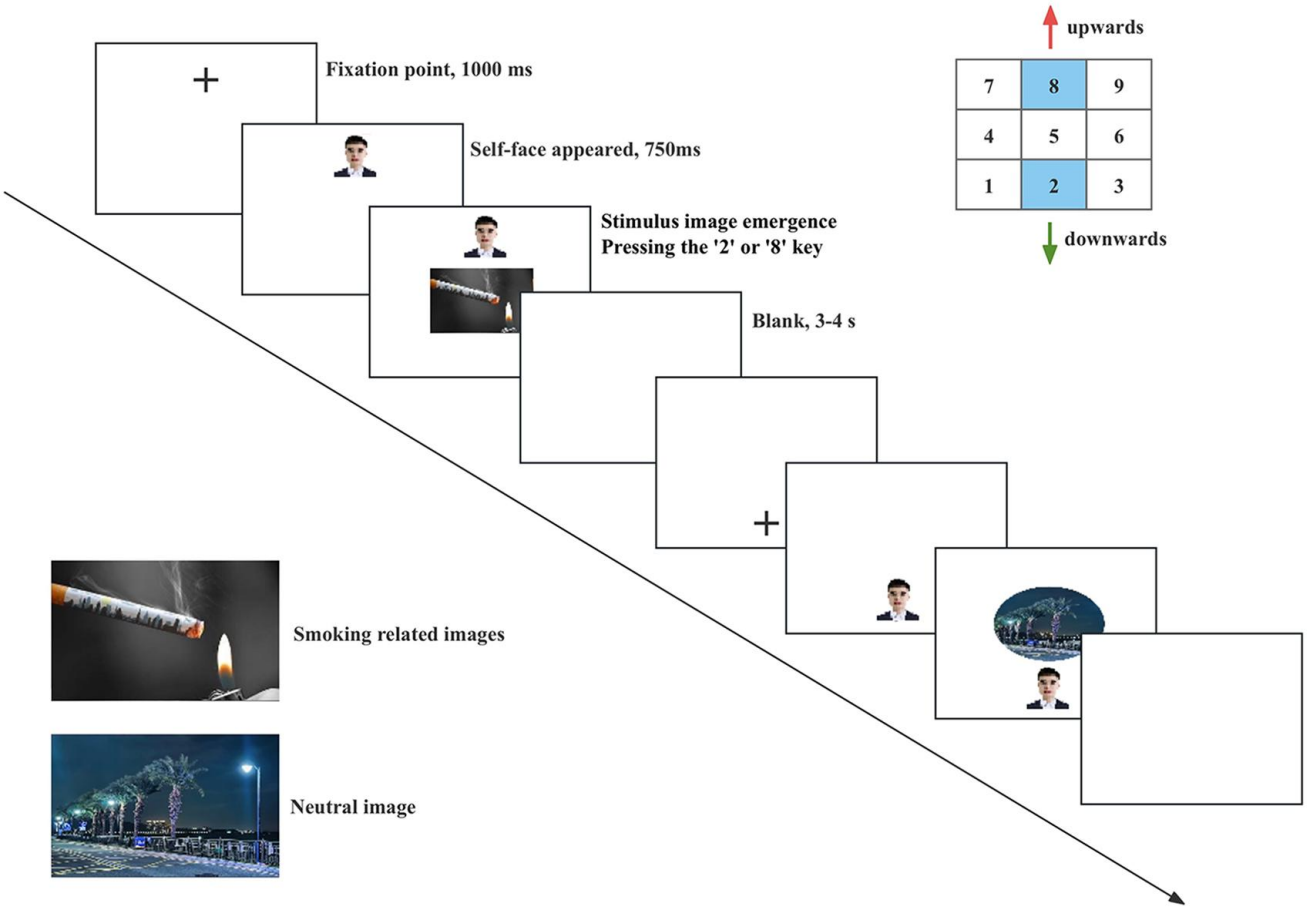
Stimulus-response compatibility task.

At the beginning of each trial, a fixation point (+) was presented randomly at the top or bottom of the screen with equal probability. The point remained on screen for 1000 ms before being replaced by a photograph of the participant. After 750 ms, a smoking-related or neutral image appeared at the center of the screen, randomly presented in either a circular or square format. Participants were instructed to either “approach circular images and avoid square images” or “approach square images and avoid circular images,” according to the block-specific instructions. Responses were made by pressing keys to control an on-screen avatar's movement toward or away from the image, simulating approach or avoidance behavior based on image shape. Participants positioned their right index finger on the “5” key as the starting point for each response, while the “2” and “8” keys were used to indicate movement direction. Pressing these keys caused the avatar to move toward or away from the stimulus image. After the movement was completed, the avatar and stimulus image disappeared, followed by a 3–4 s blank screen to minimize carryover effects from the previous trial. The experiment comprised four blocks, each containing 40 trials with an equal number of smoking-related and neutral images (20 each). Each image appeared once in a square and once in a circular format. Before the main experiment, participants completed a practice block and proceeded only after achieving at least 85% accuracy in following the keystroke instructions (Figure 2).

Only trials that met the following criteria were included in the reaction time (RT) analysis: (1) the key-press response was correct; (2) the RT fell within three standard deviations of the participant's mean; and (3) the accuracy rate reached at least 85%.

Approach bias scores were computed separately for smoking-related and neutral stimuli, following an adapted version of the method described by (27). Specifically, approach bias scores were calculated by subtracting the reaction times (RTs) of approach (pull) trials from the RTs of avoidance (push) trials. Smoking approach bias was defined as smoking avoidance RT minus smoking approach RT, and neutral approach bias was defined as neutral avoidance RT minus neutral approach RT. Thus, a positive score indicates slower avoidance responses relative to approach responses, reflecting greater difficulty in avoiding the stimulus and, consequently, a stronger approach bias. Conversely, lower or negative scores indicate reduced approach tendencies or an avoidance bias.

#### 2.4.2 fNIRS data acquisition

**Figure 3:**
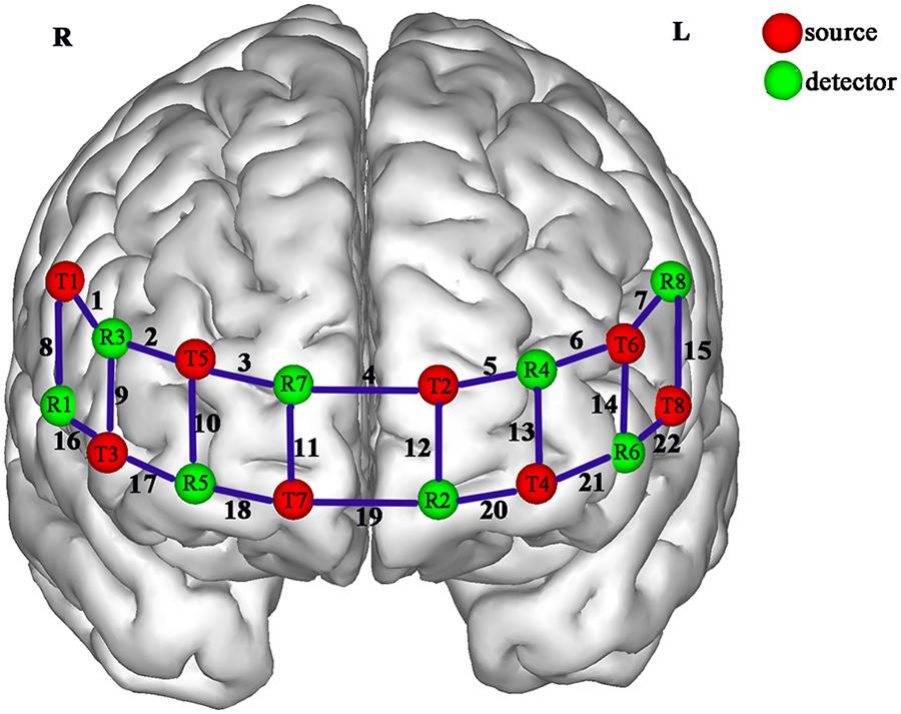
PFC channel layout (front view).

An fNIRS system (Shimadzu, Japan) was used to record hemodynamic changes in the PFC during the stimulus–response compatibility task. The system employed three wavelengths (780, 805, and 830 nm) and sampled data at 13.33 Hz. The PFC was defined as the region of interest, and participants wore an optode cap positioned according to the international 10–20 system. An 8 × 8 multichannel layout was used, consisting of eight light sources and eight detectors, forming a total of 22 channels. Optode pairs were arranged at regular intervals, with a 3 cm distance between adjacent optodes (Figure 3).

The spatial coordinates of each channel were determined using a 3D localizer (Polhemus, USA). Three-dimensional brain structures corresponding to each channel were estimated using SPM, and the associated brain regions were labeled according to the Brodmann area system (Table 3).

**Table 3:** Channels corresponding to PFC brain regions.

Brodmann area	Right area channels	Left area channels
Dorsolateral prefrontal cortex	1	7
Ventrolateral prefrontal cortex	8, 16	15, 22
Frontal polar cortex	2, 3, 4, 9, 10, 11	4, 5, 6, 12, 13, 14
Orbitofrontal cortex	17, 18, 19	19, 20, 21

### 2.5 Intervention content

Participants performed 35 min of aerobic exercise on a power bike (Monark 928E, Sweden), following the guidelines of the American College of Sports Medicine. The exercise session included a 5-minute warm-up, a 25-minute main exercise phase, and a 5-minute cool-down, during which participants maintained a constant cadence of 50 rpm. During the main exercise phase, participants cycled with their heart rate maintained within one of two target intensity ranges: 65%–75% or 75%–85% of their estimated maximum heart rate (HR_max_). Maximum heart rate was estimated using the formula: HR_max_ = 206.9−0.67 × age (28).

### 2.6 Data processing and statistical analysis

#### 2.6.1 fNIRS data processing

fNIRS data were processed using Homer2 software running on MATLAB (R2013b). Motion artifacts were identified as signal changes exceeding 10% of the standard deviation within 0.5 s. A low-pass filter (0.1 Hz) was applied to correct signal distortions caused by respiration, heartbeat, vascular pulsation, and instrument noise. A high-pass filter (0.01 Hz) was applied to remove baseline drift. Changes in optical density at the three wavelengths were converted into changes in HbO, deoxygenated hemoglobin (HbR), and total hemoglobin (HbT) concentrations within the cortical detection area using the modified Beer–Lambert law (29). Because HbO exhibits a higher signal-to-noise ratio (30), only HbO concentration data were used for subsequent analyses.

#### 2.6.2 Statistical analysis

Statistical analyses were conducted using IBM SPSS 26.0. When ANOVAs revealed significant interaction effects, *post-hoc* pairwise comparisons were performed using the LSD correction. Statistical significance was set at *p* < 0.05. Effect sizes were reported as partial eta squared (*η^2^_p_*), with *η^2^_p_* < 0.06 representing a small effect, 0.06 ≤ *η^2^_p_* < 0.14 a medium effect, and *η^2^_p_* ≥ 0.14 a large effect.

To examine differences in reaction times between the two groups under different stimulus conditions in Experiment 1, a 2 (Group: smokers, non-smokers) × 2 (Stimulus type: neutral, smoking-related) × 2 (Action: approach, avoidance) repeated-measures ANOVA was conducted. For the approach-bias scores, a 2 (Group: smokers, non-smokers) × 2 (Stimulus type: smoking-related, neutral) repeated-measures ANOVA was performed. Because the FTND scores were not normally distributed, Spearman's correlation analysis was used to examine the association between smoking approach bias and FTND scores.

To examine pre- to post-intervention changes in approach-bias scores and HbO concentration across the three groups in Experiment 2, a 3 (Group: moderate-intensity, high-intensity, control) × 2 (Stimulus type: smoking-related, neutral) × 2 (Time: pre-test, post-test) repeated-measures ANOVA was conducted.

## 3 Results

### 3.1 Approach–avoidance behavioral characteristics in smokers

**Figure 4:**
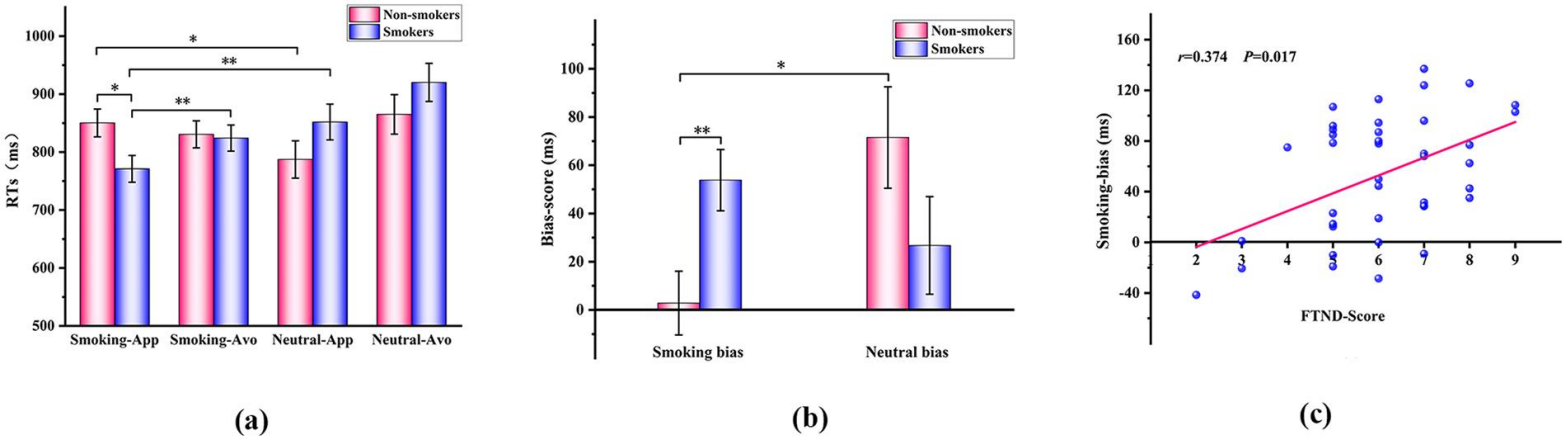
**(a)** RTs for smokers and non-smokers. **(b)** Approach-bias scores for smokers and non-smokers. **(c)** Correlation between smoking-related approach-bias scores and FTND scores. *p < 0.05; **p < 0.01. App, approach; Avo, avoidance; RTs, reaction times; FTND, Fagerström test for nicotine dependence.

A three-way repeated-measures ANOVA on RTs revealed a significant Group × Action interaction [*F_(1,75)_* = 4.460, *p* = 0.038, *η^2^_p_* = 0.056]. *post-hoc* comparisons showed that smokers responded significantly faster than non-smokers when approaching smoking-related stimuli [*F_(1,75)_* = 5.656, *p* = 0.020, *η^2^_p_* = 0.070]; smokers' RTs for approaching smoking-related stimuli were significantly faster than for approaching neutral stimuli [*F_(1,75)_* = 8.184, *p* = 0.005, *η^2^_p_* = 0.098]; non-smokers showed significantly slower RTs when approaching smoking-related stimuli compared with neutral stimuli [*F_(1,75)_* = 4.596, *p* = 0.035, *η^2^_p_* = 0.058]; and smokers responded significantly faster when approaching than avoiding smoking-related stimuli [*F_(1,75)_* = 18.231, *p* < 0.001, *η^2^_p_* = 0.095] (Figure 4a; Table 4).

**Table 4:** Reaction time results for approach and avoidance behaviors.

Group	Smoking		Neutral	
	Approach	Avoidance	Approach	Avoidance
Non-Smokers	850.38 ± 24.04	830.7 ± 23.36	787.31 ± 32.2	865.17 ± 34.16
Smokers	771.07 ± 23.12	824.22 ± 22.46	852.01 ± 30.97	920.27 ± 32.85

A two-way repeated-measures ANOVA on approach-bias scores revealed a significant group × stimulus type interaction [*F*_(1,75)_ = 5.651, *p* = 0.020, *η^2^_p_* = 0.070]. *Post-hoc* comparisons indicated that smokers exhibited a significantly greater approach bias toward smoking-related stimuli than non-smokers [*F*_(1,75)_ = 7.723, *p* = 0.007, *η^2^_p_* = 0.093], while non-smokers showed a significantly greater approach bias toward neutral stimuli than toward smoking-related stimuli (Figure 4b; Table 5).

**Table 5:** Approach bias scores.

Group	Smoking bias	Neutral bias
Non-Smokers	2.86 ± 13.22	71.53 ± 21.04
Smokers	53.83 ± 12.71	26.76 ± 20.24

Furthermore, correlation analysis revealed a significant positive association between smokers' approach-bias scores for smoking-related stimuli and their FTND scores (r = 0.374, *p* = 0.017) (Figure 4c).

### 3.2 Behavioral effects of exercise interventions on approach bias

**Figure 5:**
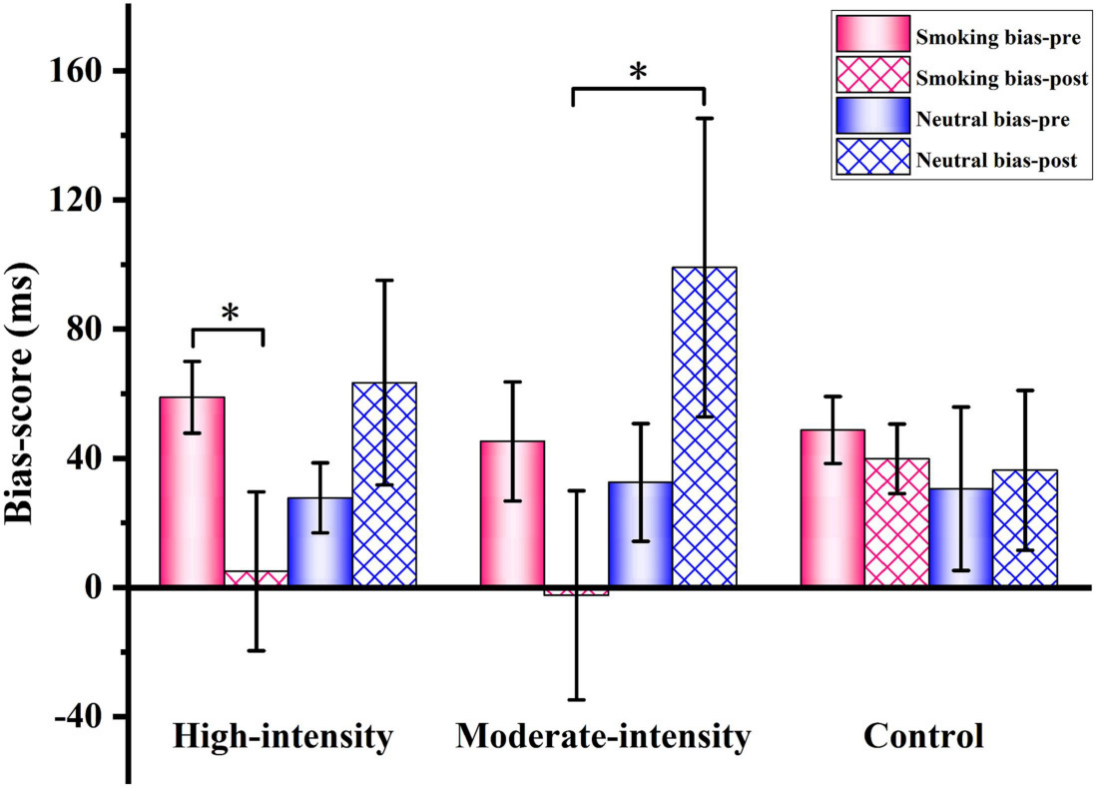
Bias scores of smokers before and after exercises of different intensities. *p < 0.05; **p < 0.01. pre, pre-test; post, post-test.

A three-way repeated-measures ANOVA on approach-bias scores revealed a significant Group × Time × Stimulus Type interaction [*F_(2,54)_* = 4.163, *p* = 0.046, *η^2^_p_* = 0.061]. Additionally, a significant Stimulus Type × Time interaction was observed [*F_(2,54)_* = 4.955, *p* = 0.030, *η^2^_p_* = 0.084], along with a significant main effect of Stimulus Type [*F_(1,54)_* = 5.708, *p* = 0.019, *η^2^_p_* = 0.071]. *Post-hoc* comparisons indicated that the high-intensity exercise group exhibited a significant reduction in smoking-related approach-bias scores from pre- to post-exercise [*F_(1,54)_* = 4.466, *p* = 0.044, *η^2^_p_* = 0.073]. The moderate-intensity exercise group showed no significant differences in approach-bias scores between the two stimulus types before exercise; however, following exercise, approach bias toward smoking-related stimuli was significantly lower than that toward neutral stimulus [*F_(1,54)_* = 4.219, *p* = 0.045, *η^2^_p_* = 0.072]. In contrast, the control group showed no significant changes in approach-bias scores from pre- to post-intervention (Figure 5; Table 6).

**Table 6:** Approach bias scores across different images in three groups of smokers pre- and post-intervention.

Group	Smoking bias		Neutral bias	
	Pre-test	Post-test	Pre-test	Post-test
High intensity exercise (*n* = 20)	58.90 ± 11.10	4.98 ± 24.61	27.78 ± 10.86	63.43 ± 31.65
Moderate intensity exercise (*n* = 17)	45.26 ± 18.43	−2.44 ± 32.34	32.56 ± 18.22	99.06 ± 46.21
Control (*n* = 20)	48.75 ± 10.34	39.85 ± 10.8	30.55 ± 25.29	36.3 ± 24.73

### 3.3 Neural effects of exercise interventions

**Figure 6:**
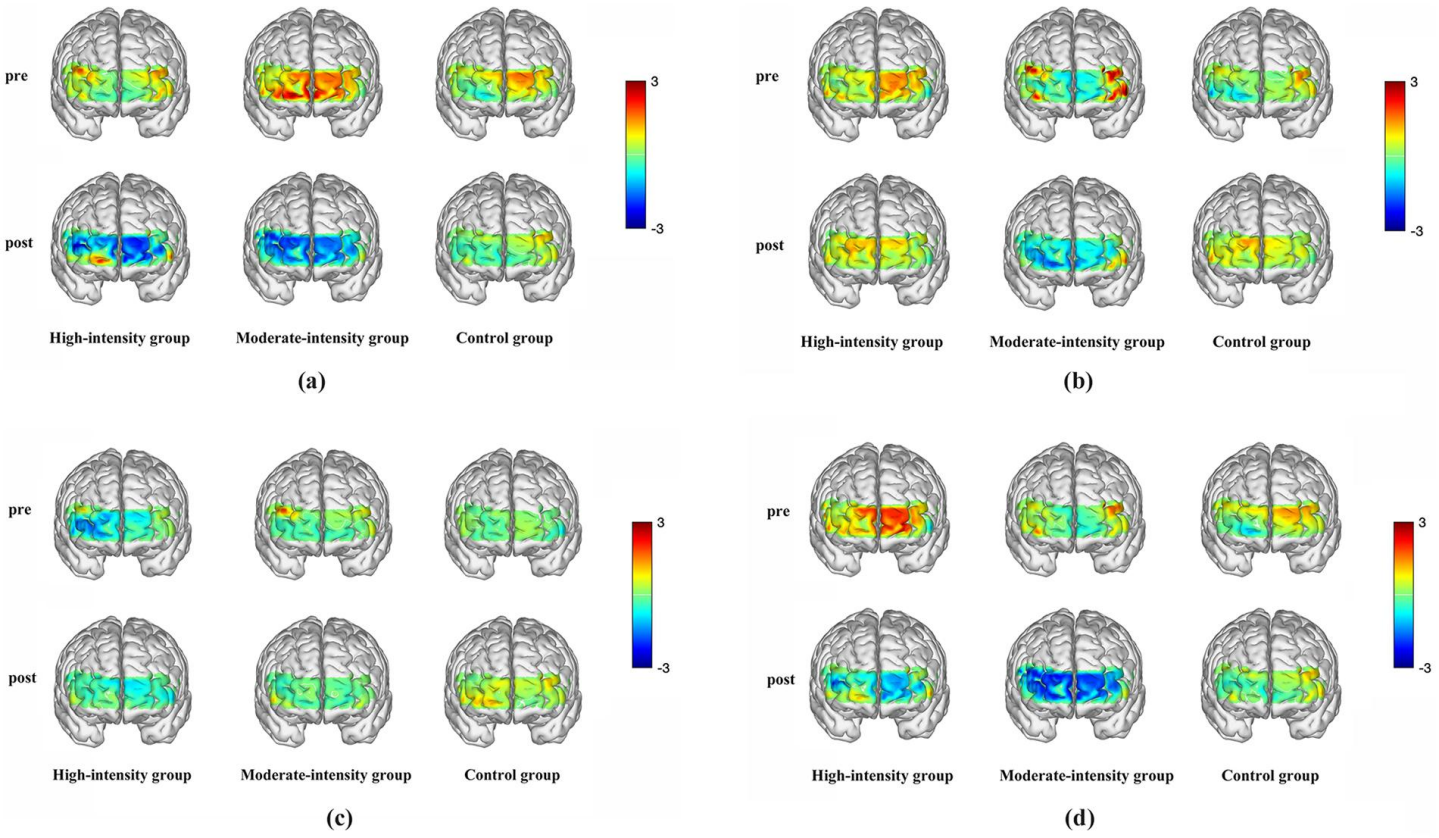
Mean PFC HbO concentrations before and after the intervention among the three participant groups under different stimuli. **(a)** Smoking-Approach; **(b)** Smoking-Avoidance; **(c)** Neutral-Approach; **(d)** Neutral-Avoidance. The color bar represents relative changes in HbO concentration. Higher values indicate greater HbO concentration, while lower values indicate reduced HbO concentration.

Analysis of participants' HbO responses showed a significant main effect of time in the R-OFC [*F_(1,54)_* = 11.473, *p* = 0.011, *η^2^_p_* = 0.206] and a significant group × time × stimulus-type interaction [*F_(2,54)_* = 4.231, *p* = 0.029, *η^2^_p_* = 0.177]. Simple-effects analyses further indicated that during smoking-approach responses, post-exercise HbO levels were significantly lower than pre-exercise levels in both the high-intensity [*F_(1,54)_*= 13.573, *p* < 0.001, *η^2^_p_* = 0.182] and moderate-intensity [*F_(1,54)_* = 10.628, *p* = 0.006, *η^2^_p_* = 0.203] exercise groups (Figure 6; Tables 7, 8).

**Table 7:** Hbo concentration changes during smoking-related tasks before and after intervention.

ROI	Group	Smoking-Approach		Smoking-Avoidance	
		Pre-test	Post-test	Pre-test	Post-test
R-DLPFC	High intensity exercise	1.95 ± 1.42	1.80 ± 0.36	0.12 ± 0.25	−1.08 ± 0.44
	Moderate intensity exercise	0.63 ± 0.31	0.43 ± 0.19	5.55 ± 2.88	−1.83 ± 0.76
	Control	0.59 ± 0.27	1.14 ± 0.52	1.81 ± 0.86	−0.38 ± 0.28
L-DLPFC	High intensity exercise	1.80 ± 0.98	0.00 ± 0.22	1.94 ± 1.41	1.41 ± 0.36
	Moderate intensity exercise	1.05 ± 0.37	−0.58 ± 0.44	2.04 ± 1.47	1.24 ± 0.29
	Control	1.13 ± 0.41	1.61 ± 0.56	1.19 ± 0.32	0.46 ± 0.26
R-VLPFC	High intensity exercise	1.30 ± 0.39	0.25 ± 0.28	0.52 ± 0.28	−0.63 ± 0.33
	Moderate intensity exercise	1.24 ± 0.42	−1.26 ± 0.51	3.05 ± 0.72	−1.24 ± 0.49
	Control	0.94 ± 0.33	0.66 ± 0.35	1.61 ± 0.44	0.29 ± 0.25
L-VLPFC	High intensity exercise	1.16 ± 0.37	1.09 ± 0.40	−0.29 ± 0.22	0.00 ± 0.21
	Moderate intensity exercise	0.25 ± 0.25	−0.02 ± 0.18	3.38 ± 1.75	1.11 ± 0.38
	Control	0.07 ± 0.21	0.94 ± 0.46	1.08 ± 0.36	−0.04 ± 0.19
R-FPC	High intensity exercise	0.53 ± 0.27	−1.45 ± 0.56	0.45 ± 0.24	1.13 ± 0.42
	Moderate intensity exercise	1.02 ± 0.38	−1.95 ± 0.61	−0.15 ± 0.23	−0.76 ± 0.34
	Control	0.47 ± 0.30	0.03 ± 0.17	0.13 ± 0.21	0.91 ± 0.37
L-FPC	High intensity exercise	0.47 ± 0.26	−1.24 ± 0.52	1.11 ± 0.33	0.25 ± 0.23
	Moderate intensity exercise	0.97 ± 0.35	−1.27 ± 0.48	0.53 ± 0.22	−0.70 ± 0.30
	Control	1.06 ± 0.40	0.38 ± 0.25	0.43 ± 0.20	0.56 ± 0.27
R-OFC	High intensity exercise	0.24 ± 0.20	−0.28 ± 0.29*	1.23 ± 0.34	0.45 ± 0.27
	Moderate intensity exercise	2.77 ± 1.73	−1.48 ± 0.58**	1.17 ± 0.31	−1.47 ± 0.55
	Control	−0.10 ± 0.23	−0.01 ± 0.22	−0.16 ± 0.22	0.55 ± 0.29
L-OFC	High intensity exercise	0.55 ± 0.29	−2.02 ± 0.97	1.15 ± 0.29	0.55 ± 0.28
	Moderate intensity exercise	2.49 ± 1.70	−1.76 ± 0.62	0.94 ± 0.26	−0.29 ± 0.23
	Control	0.40 ± 0.28	−0.06 ± 0.24	0.22 ± 0.19	0.48 ± 0.25

DLPFC, dorsolateral prefrontal cortex; VLPFC, entrolateral prefrontal cortex; FPC, frontal polar cortex; OFC, orbitofrontal cortex; L and R, left and right hemispheres.

* *p* < 0.05.

** *p* < 0.01.

**Table 8:** Hbo concentration changes during neutral tasks before and after intervention.

ROI	Group	Neutral-Approach		Neutral-Avoidance	
		Pre-test	Post-test	Pre-test	Post-test
R-DLPFC	High intensity exercise	1.25 ± 0.41	−0.94 ± 0.37	−0.17 ± 0.24	1.69 ± 0.45
	Moderate intensity exercise	0.49 ± 0.26	0.22 ± 0.21	1.09 ± 0.37	−0.68 ± 0.30
	Control	−0.85 ± 0.39	−0.01 ± 0.23	1.34 ± 0.42	2.27 ± 0.71
L-DLPFC	High intensity exercise	0.50 ± 0.29	0.17 ± 0.22	1.02 ± 0.36	0.55 ± 0.29
	Moderate intensity exercise	1.46 ± 0.52	0.24 ± 0.20	2.06 ± 0.58	−0.62 ± 0.27
	Control	0.04 ± 0.24	0.78 ± 0.35	1.05 ± 0.33	1.42 ± 0.49
R-VLPFC	High intensity exercise	0.43 ± 0.27	−0.60 ± 0.33	0.91 ± 0.32	0.57 ± 0.28
	Moderate intensity exercise	0.56 ± 0.30	−0.23 ± 0.25	1.75 ± 0.50	−1.82 ± 0.56
	Control	0.08 ± 0.22	1.10 ± 0.42	1.40 ± 0.41	0.85 ± 0.37
L-VLPFC	High intensity exercise	0.08 ± 0.19	−1.50 ± 0.58	−0.34 ± 0.23	1.10 ± 0.38
	Moderate intensity exercise	0.78 ± 0.34	0.06 ± 0.22	0.89 ± 0.34	−0.02 ± 0.22
	Control	−0.44 ± 0.28	1.06 ± 0.39	0.67 ± 0.30	1.11 ± 0.40
R-FPC	High intensity exercise	−1.05 ± 0.46	−0.56 ± 0.31	1.04 ± 0.33	−0.57 ± 0.28
	Moderate intensity exercise	0.13 ± 0.21	−0.19 ± 0.23	0.04 ± 0.21	−2.13 ± 0.63
	Control	0.00 ± 0.20	0.33 ± 0.27	0.45 ± 0.24	−0.07 ± 0.23
L-FPC	High intensity exercise	−0.16 ± 0.24	−0.59 ± 0.34	1.18 ± 0.35	−0.75 ± 0.31
	Moderate intensity exercise	0.20 ± 0.22	0.03 ± 0.21	0.35 ± 0.22	−1.56 ± 0.51
	Control	0.05 ± 0.20	0.62 ± 0.30	0.96 ± 0.33	0.39 ± 0.26
R-OFC	High intensity exercise	−0.54 ± 0.29	0.11 ± 0.22	1.26 ± 0.37	−0.31 ± 0.24
	Moderate intensity exercise	−0.44 ± 0.26	0.08 ± 0.20	0.65 ± 0.26	−1.93 ± 0.54
	Control	−0.31 ± 0.25	0.77 ± 0.33	−0.36 ± 0.23	0.19 ± 0.21
L-OFC	High intensity exercise	−0.28 ± 0.23	0.05 ± 0.20	1.60 ± 0.48	−1.38 ± 0.52
	Moderate intensity exercise	−0.58 ± 0.28	0.19 ± 0.23	0.07 ± 0.20	−1.81 ± 0.58
	Control	−0.17 ± 0.24	−0.03 ± 0.21	0.23 ± 0.22	−0.11 ± 0.24

## 4 Discussion

This study demonstrated that smokers show faster reaction times and stronger approach biases toward smoking-related cues than non-smokers, and these biased responses were strongly associated with nicotine dependence. In addition, aerobic exercise significantly modulated the cognitive processes underlying this bias. Exercise not only reduced behavioral approach bias scores but also decreased PFC activation in response to smoking cues. These findings suggest that exercise may attenuate cue-induced bias by enhancing prefrontal regulatory function.

According to the dual-processing theory of addiction, two distinct information-processing systems operate in individuals with substance dependence: a fast-acting impulsive system and a reflective system that relies on higher-order cognitive control (31, 32). These two systems may process information either synergistically or in competition with one another. Given the rewarding properties of smoking-related cues for smokers, the impulsive system tends to dominate when processing these stimuli. As a result, smoking cues are processed more rapidly, whereas the reflective system shows diminished inhibitory control due to insufficient cognitive resource allocation (33). Consequently, smokers display faster responses to smoking-related cues and exhibit a pronounced approach bias toward them. Importantly, this cognitive bias is positively associated with the severity of nicotine dependence. Individuals with different levels of dependence respond differently to the same cues, and such approach tendencies—shaped by long-term dependence—may further enhance subsequent substance use, thereby exacerbating nicotine dependence. These findings further highlight the high reward value that smoking-related stimuli hold for smokers (34, 35). In contrast, non-smokers showed a significantly greater approach bias toward neutral images than toward smoking-related cues. This difference may reflect distinct neural pathways underlying stimulus processing in smokers vs. non-smokers, resulting in different processing speeds for the same visual stimuli (13, 36).

Intervention studies have demonstrated that exercise can effectively modulate the cognitive biases exhibited by smokers. Previous research has shown that exercise interventions can improve cognitive processes related to executive control and reward evaluation, thereby alleviating cognitive impairments in individuals attempting to quit and reducing tobacco dependence (37). Compared with long-term exercise interventions, which primarily reflect cumulative training adaptations, acute exercise paradigms are better suited to examining the immediate modulatory effects of exercise on cognitive processing (38, 39). Such paradigms allow researchers to capture transient, state-dependent changes induced by exercise within a short time frame while minimizing potential confounding factors such as training adherence and long-term lifestyle changes. The mechanisms through which exercise attenuates withdrawal symptoms and cognitive biases may involve the temporary allocation of additional cognitive resources and a reduction in attentional capture elicited by smoking-related cues. According to Selective Attention Bias Theory (40, 41), when smoking behavior is restricted, smokers tend to automatically allocate more cognitive resources toward smoking-related cues, resulting in heightened attentional and approach biases. Within this theoretical framework, acute aerobic exercise may transiently enhance top-down cognitive control, thereby disrupting this automatic bias process and reducing the influence of smoking cues on behavioral responses. The behavioral results of the present study provide direct support for this perspective. Our findings revealed that, following acute aerobic exercise, smokers exhibited marked changes in their responses to smoking-related cues, including reduced approach bias scores, faster avoidance responses, and diminished approach tendencies. These results indicate that even a single session of exercise can produce measurable modulatory effects on smoking-related cognitive biases, suggesting that the impact of exercise on addiction-related cognitive processing does not entirely depend on the stable adaptations induced by long-term training, but can instead manifest rapidly through the immediate cognitive regulatory effects elicited by acute exercise.

The HbO results offer neural-level support for the behavioral mechanisms described above. Before the intervention, smoking-related tasks elicited broadly elevated PFC activation, reflecting greater cognitive load and heightened reward sensitivity when smokers processed smoking-related cues. After the intervention—particularly following high-intensity exercise—HbO levels in the right OFC significantly decreased, suggesting that exercise attenuated the OFC's reward-related processing of smoking cues and reduced cue-induced salience. This decrease in activation corresponded to the diminished approach bias, indicating that exercise may enhance the efficiency of prefrontal inhibitory control and reduce smokers' propensity to overreact to smoking-related cues. Regular exercise has been shown to increase cerebral blood flow and upregulate brain-derived neurotrophic factor (BDNF) expression (42, 43). BDNF supports the repair of nicotine-damaged neurons (44), facilitates improvements in smokers' decision-making processes (45, 46), and strengthens inhibitory control over smoking urges (45). Aerobic exercise also stimulates the release of dopamine and other neurotransmitters (47, 48), providing an alternative source of reward that may partially substitute for the reinforcing effects of smoking (9, 49). Such neurochemical changes may help restore the functioning of neural circuits commonly disrupted by addictive substances, thereby mitigating smoking-related damage to prefrontal reward-processing systems (50). Moreover, exercise may help smokers regulate stress responses by modulating cortisol secretion, reducing the effects of chronic stress and decreasing their tendency to automatically seek nicotine as a coping strategy (51, 52).

While the present study focused on aerobic exercise, it is important to note that different forms of physical activity (such as resistance exercise) may exert distinct effects on cognitive control and reward-related neural circuits. Evidence from prior research suggests that aerobic exercise preferentially enhances prefrontal-mediated executive control and dopaminergic reward regulation (53), whereas resistance exercise may more strongly influence muscular strength and metabolic adaptations, potentially modulating cognitive function through alternative neuroendocrine pathways (54). Although direct comparisons between aerobic and resistance exercise were beyond the scope of the current study, the observed dose-dependent effects of high- vs. moderate-intensity aerobic exercise on HbO and approach bias highlight that exercise intensity can differentially impact neural and behavioral outcomes. Future studies should directly investigate these differential effects to optimize exercise-based interventions for attenuating cue-induced bias in smokers.

Despite supporting the modulatory effect of exercise on smokers' approach bias, several limitations should be noted. Smoking status was primarily assessed using the FTND, without biochemical verification, which may limit the precision of characterizing recent nicotine exposure. The use of a resting control condition may have introduced expectancy effects, as participants in the exercise group could anticipate cognitive or health-related benefits. Moreover, the sample consisted exclusively of healthy male participants, which may limit the generalizability of the findings. Future studies should recruit more representative samples, including females and individuals with comorbidities, and incorporate objective biomarkers and active control conditions to further clarify the mechanisms underlying exercise-induced modulation of approach bias. Despite these limitations, our findings provide evidence that acute aerobic exercise can transiently modulate approach bias toward smoking cues, supporting the potential utility of exercise-based interventions in smoking cessation.

## 5 Conclusion

Smokers display a pronounced behavioral approach bias toward smoking-related cues, which is positively associated with the severity of nicotine dependence. This finding suggests that smoking-related cues engage the brain's reward circuitry, facilitating rapid, automatic responses. Acute aerobic exercise effectively attenuates this bias, as reflected by reductions in approach behavior and decreased HbO concentration in the PFC. These results indicate that exercise temporarily strengthens cognitive control over addiction-related cues and helps restore PFC function compromised by nicotine exposure.

## Data Availability

The original contributions presented in the study are included in the article/Supplementary Material, further inquiries can be directed to the corresponding authors.
